# Adsorptive removal of organophosphate pesticides from aqueous solution using electrospun carbon nanofibers

**DOI:** 10.3389/fchem.2024.1454367

**Published:** 2024-08-26

**Authors:** Bukola O. Adesanmi, Shobha Mantripragada, Raphael D. Ayivi, Panesun Tukur, Sherine O. Obare, Jianjun Wei

**Affiliations:** ^1^ Department of Nanoscience, Joint School of Nanoscience and Nanoengineering, University of North Carolina, Greensboro, NC, United States; ^2^ Department of Nanoengineering, Joint School of Nanoscience and Nanoengineering, North Carolina Agricultural and Technical State University, Greensboro, NC, United States

**Keywords:** adsorption, removal, organophosphate pesticides, electrospinning, carbon nanofibers, environmental remediation

## Abstract

Organophosphate pesticides (OPPs) are widely prevalent in the environment primarily due to their low cost and extensive use in agricultural lands. However, it is estimated that only about 5% of these applied pesticides reach their intended target organisms. The remaining 95% residue linger in the environment as contaminants, posing significant ecological and health risks. This underscores the need for materials capable of effectively removing, recovering, and recycling these contaminants through adsorption processes. In this research, adsorbent materials composed of electro-spun carbon nanofibers (ECNFs) derived from polyacrylonitrile was developed. The materials were characterized through several techniques, including scanning electron microscopy (SEM), X-ray photoelectron spectroscopy (XPS), Fourier Transform Infrared Spectroscopy (FTIR), Brunauer–Emmett–Teller (BET) analysis, and contact angle measurements. SEM analysis revealed details of the structural properties and inter-fiber spacing variations of the carbon nanofibers. The results revealed that ECNFs possess remarkable uniformity, active surface areas, and high efficiency for adsorption processes. The adsorption studies were conducted using batch experiments with ethion pesticide in aqueous solution. High-Performance Liquid Chromatography–Diode Array Detector (HPLC-DAD) was utilized to quantify the concentrations of the OPP. Various parameters, including adsorbent dosage, pH, contact time, and initial ethion concentration, were investigated to understand their impact on the adsorption process. The adsorption isotherm was best described by the Freundlich model, while the kinetics of adsorption followed a non-integer-order kinetics model. The adsorption capacity of the ECNFs for OPP removal highlights a significant advancement in materials designed for environmental remediation applications. This study demonstrates the potential of ECNFs to serve as effective adsorbents, contributing to the mitigation of pesticide contamination in agricultural environments.

## 1 Introduction

Organophosphate pesticides (OPPs) are toxic chemicals and one of the most widely used pest-controlling chemicals in agriculture. They account for approximately 40% of all pesticides produced and used commercially around the world, OPPs account for about 70% of total pesticides used in the United States (Dehghani et al., 2017; Mehta et al., 2022; Dissanayake et al., 2019). Their large usage generates significant amount of OPP residues, and these residues remain as contaminants in the soil, water, air, and food endangering all living strata exposed to these chemicals in various ways (Mali et al., 2022; Kaushal, Khatri, and Arya, 2021). When these pesticides are exposed to living beings, the major toxicological effect is the irreversible inhibition of the acetylcholinesterase (AChE) enzyme, which is involved in signal neurotransmission, and thus its inhibition causes impairment of the respiratory tract and neuromuscular transmission (Kaushal, Khatri, and Arya, 2021; Mulla et al., 2020; Chawla et al., 2018). OPP contamination has been extensively documented in agricultural soils, water bodies, and industrial regions across the globe. This pervasive issue affects many countries and continents, including but not limited to Brazil, China, Thailand, India, Mexico, Nepal, Bangladesh, Japan and Africa (Mali et al., 2022; Ore et al., 2023; Velasco et al., 2014; Derbalah et al., 2019; Sumon et al., 2018; Zhang et al., 2021). Most of these regions have experienced significant levels of pesticide residues above regulatory threshold in their environments, highlighting a widespread problem that impacts both human health and ecological systems. A comprehensive analysis and risk assessment were conducted on a variety of water bodies, soil and sediment samples collected from these regions to evaluate potential environmental and health impacts. For instance, OPP contamination was reported in the agricultural soils of China, the study specifically detected and quantified high concentrations of over nine different OPPs. The concentrations were found to be notably high which highlights a significant level of OPP contamination in the region, posing potential risks to both environmental and human health (Pan et al., 2018; Bhandari et al., 2020). Similarly, the aquatic risk assessment of OPPs in both surface water and sediments was performed on two different water systems in Bangladesh. The findings revealed that the OPPs pose a significant risk to aquatic life in these regions (Sumon et al., 2018). The predominant contamination and distribution of OPPs in the environment have also been reported in several parts of Africa. A recent report highlighted human exposure to OPPs through inhalation and soil ingesting by investigating the concentration of OPPs in soil and air samples from two different sites in South Africa (Degrendele et al., 2022). Despite the hazards that these OPPs present, they form an indispensable part of modern agriculture and has raised global health concerns.

Organophosphorus pesticides can be removed from the environment using several techniques, such as photocatalysis, biochemical decomposition, electrochemical decomposition, separation using various membranes, oxidation, and adsorption (Dehghani et al., 2019; Mehta et al., 2022). The adsorption of pesticides onto various materials is one of the most promising methods for removing them from water due to its efficiency, affordability, and sustainability capacity. Several adsorbents’ materials such as activated carbon, multi walled-carbon nanotubes, graphene, magnetic materials, metal organic framework and gold nanomaterials have been used for the removal of organophosphate pesticides (Wanjeri et al., 2018; Momić et al., 2016; Dehghani et al., 2019; Zhu et al., 2015; Alrefaee et al., 2023; Seif et al., 2015). These studies reported various adsorption capacities ranging from 10 to 300 mg/g. Among these materials, carbon-based nanomaterials have been reported to possess excellent adsorption efficiency due to the molecular interactions, large specific surface area, good adsorption capacity and mechanical strength. Most of these carbon-based materials are functionalized with other nanoparticles which are dispersed into aqueous solution containing the organophosphate pesticides (Wanjeri et al., 2018; Sereshti et al., 2023; Mahpishanian, Sereshti, and Baghdadi, 2015; Uddin, 2021). However, this dispersion poses a major concern for the adsorbent removal procedure. Most research used magnetic separation, centrifugation, and ultra filtration approaches to get rid of the adsorbent after the adsorption process (Wanjeri et al., 2018; Dehghani et al., 2021; Alrefaee et al., 2023; Mahpishanian, Sereshti, and Baghdadi, 2015; Firozjaee et al., 2017; Sarno et al., 2017). These removal methods limit the real-world applications. Another challenging issue from these adsorbents is the cost-effectiveness because these materials are very expensive to produce on a large-scale basis and limited availability of materials. As a result, it is vital to develop simple and effective alternative materials such as carbon nanofiber that are easy to remove OPPs.

Nanofiber-based scaffolds are known for their inexpensive production and are typically synthesized using one of three primary methods: phase separation, self-assembly, and electrospinning. Each method has its own advantages, but electrospinning is commonly employed in the field. This preference is largely because electrospinning offers superior control over several critical properties of the nanofibers, including their diameter, alignment, and the ability to encapsulate various signals within the fibers. The versatility and precision of the electrospinning technique make it the most widely utilized method for fabricating nanofiber scaffolds (Kim et al., 2016). Moreover, the fabrication of fibers with sizes ranging from sub-microns to nanometers can be done simply and effectively using the electrospinning technology (Zhou et al., 2010). Technological breakthroughs have made it possible to industrialize electrospinning, which makes carbon fibers manufacture and real-world applications practical (Mokhena et al., 2022). Electro-spun carbon nanofibers (ECNFs) have gained interest recently and have been employed in water treatment due to their porosity, high surface areas, fast adsorption rate, ease of functionalization, inexpensive cost, and nanoscale diameter (Thamer et al., 2019; Mantripragada et al., 2020). These properties make them advantageous over traditional adsorbents. The hydrophobic surface, micrometer-scale inter-fiber pores, good stability, ease of collection and regeneration make it a good adsorbent material for OPPs (Zhu et al., 2023). The ECNFs are graphitic thread-like materials derived from electrospinning of organic polymer such as polyacrylonitrile, polymethyl methacrylate, polyvinyl alcohol, starch, polyamide, cellulose, etc., followed by heat treatment with high carbon content yield and mechanical strength (Maddah et al., 2017; Ibupoto et al., 2018).

The orientation of electrospun carbon nanofibers (E-CNFs) significantly influences their interactions with various molecules. This orientation can be adjusted based on the design of the electrospinning process and the intended application. Numerous studies have investigated the impact of E-CNF orientation and alignment on their interaction with cells (Huang et al., 2016; Kim et al., 2016; Gui et al., 2019). For example, one recent study examined the effect of fiber orientation on cell adhesion by imaging cell growth on polyvinyl alcohol (PVA)-gelatin scaffolds. The results showed that cells on randomly oriented fibers exhibited an isotropic morphology with indistinct borders between the cells and nanofibers. In contrast, cells on aligned fibers displayed a uniformly stretched linear growth pattern, suggesting that fiber orientation can impact cellular behavior (Huang et al., 2016). This study aims to investigate how fiber orientation affects the permeability and absorptivity of E-CNFs. By comparing both Randomly oriented E-CNFs (R-ECNFs) and Aligned oriented E-CNFs (A-ECNFs). R-ECNFs are typically synthesized with a lower collector rotation speed, usually less than 500 rpm, and sometimes using different collectors or electrospinning setups. On the other hand, (A-ECNFs) are collected at higher rotation speeds, often exceeding 1,000 rpm. This distinction in production methods leads to variations in fiber alignment, which in turn affects the material’s properties and interactions with other molecules.

This paper focuses on the use of ECNFs for adsorptive removal of OPPs from aqueous solution, specifically demonstrates an efficient and reusable ECNFs for the removal of ethion pesticide. The concentration of ethion in water samples was measured using high performance liquid chromatography diode array detector (HPLC-DAD). The effects of solution pH, OPP concentration, adsorbent dosage as well as contact time on the adsorption of ethion were investigated. The adsorption isotherm and kinetics were examined and discovered. The recyclability of the material as well as applicability for real-world samples were also studied. The results showed advantages of its reusability and ease of separation of OPPs from aqueous solution, making it a very prominent, affordable, and environmentally friendly material. While there are reports on adsorption of OPPs using various carbon-based adsorbents, to the best of our knowledge, there are few reports on the removal of OPPs using activated carbon nanofiber.

## 2 Materials and methods

### 2.1 Chemicals and materials

Polyacrylonitrile (PAN, MW = 150,000), N,N-dimethyl formamide (DMF), HPLC grade methanol, Ethion (98% analytical grade), hydrochloric acid, and sodium hydroxide were all purchased from Sigma Aldrich. All chemicals were used as received without further purification. Stock standard solution (1,000 mg/mL) of ethion was prepared in methanol and stored at 4°C. The working solutions were prepared daily by diluting with DI water.

### 2.2 Preparation of carbon nanofiber

The selection of DMF as the electrospinning solvent was based on its suitable boiling point and electrical conductivity which is completely evaporated during spinning and subsequent heat process (Awad et al., 2021). In comparison to other carbon polymer precursors, polyacrylonitrile (PAN) was utilized because of its high strength, slow rate of weight loss during carbonization and a higher carbon yield, making it a superior carbon precursor for producing ECNFs (Mahar et al., 2019; Zhang et al., 2014).

The preparation of A-ECNFs was according to our previously reported methods and process (Liu et al., 2018; Allado et al., 2021; Yin et al., 2022), while the R-ECNFs were obtained according to the previous method (Zeng et al., 2017) with reduced spinning speed. We believe spinning speed of collector plays a key role determining the orientation of the fibers depending on if the formation of fiber matches rotating rate of the collector. Briefly, 10% (w/v) Polyacrylonitrile (PAN) was dissolved in N, N-dimethylformamide (DMF) and stirred at room temperature for 24 h. The solution was filled into a 10 mL syringe attached to an 18-gauge needle tip. The solution was then electro-spun onto an aluminum foil wrapped collector at the rate of 400 revolutions per minute (rpm) for random fibers. The aligned fibers were electro-spun onto a fast-rolling collector at the rate of 2,000 rpm. A high voltage of 15 KV was applied and the distance between the needle tip and the collector was maintained at 15 cm apart for both fiber production. The fibers were electro-spun for 5 h with a flow rate of 1 mL/h precursor solution. After electrospinning, the obtained fibers were peeled from the aluminum foil prior to the stabilization treatment.

The prepared PAN nanofibers were stabilized in a muffle furnace under air at 280°C for 6 h at a heating rate of 1°C/min. This process was followed by a carbonization process. Specifically, the brownish color stabilized PAN nanofibers were carbonized at 1,200°C for 1 h with a heating rate of 5°C min^−1^ under a nitrogen atmosphere to improve the mechanical strength. The ECNFs were washed with DI water and dried at 80°C for 3–4 h.

### 2.3 Batch adsorption experiments

The adsorption experiments were performed using a batch technique in an aqueous solution at room temperature in a glass vial. The experiments were carried out using 10 mL ethion solution with a stirring speed of 160 rpm. All experiments were done in triplicates. The adsorbent dosage, ethion concentration, pH, and time were changed as variable factors. The parameters influencing the adsorption were optimized by varying one parameter while keeping other parameters constant. The optimum dosage was selected by testing two nanofiber masses 2 mg and 5 mg, respectively. The influence of pH was studied by adjusting the solution pH from 2 to 9 using 0.1 M NaOH and 0.1 M HCl. The time interval was varied from 0 to 180 min at 15 min intervals. The effect of ethion concentration was investigated by varying the concentration form 5 mg/L to 30 mg/L. To analyze the residual ethion concentration, the fiber material was taken out of the solution and ethion sample was moved using a pipette into the 1 mL glass vial for HPLC analysis. The percentage removal of ethion and the adsorption capacity (mg/g) of the fibers at the equilibrium and time t, were calculated using Equations 1–3:

$$\text{Removal}\;\text{efficiency}\left(\%\right)=\left(\frac{C_{o}-C_{e}}{C_{o}}\right)\times 100$$ (1)

$$q_{e}=\left(\frac{C_{o}-C_{t}}{m}\right)V$$ (2)

$$q_{t}=\left(\frac{C_{o}-C_{t}}{m}\right)V$$ (3)

where *Co* is the initial concentration of ethion, *Ce* is the final concentration of ethion, *Ct* is the concentration of ethion at time t, *q*_*e*_ and *qt* is the amount of OPP adsorbed at equilibrium and time t (min), respectively; V is the volume of the solution (L), and m is the weight of the adsorbent (g).

The recyclability test was performed by soaking the used ECNFs in 5 mL methanol for 2 h. The fibers were then removed and washed with distilled water three times, then dried in the oven at 80°C before the next use. The concentration of ethion was measured using HPLC-DAD. The analytical column of the HPLC was C18 column (50 mm × 2.1 m, 1.9 µm), the mobile phase was acetonitrile and 0.03 M phosphoric acid in 80:20 ratio. The solvent blend was isocratic. The flow rate was maintained at 0.2 mL min^−1^ and the injection volume was 20 µL. The wavelength of the detector was fixed at 254 nm after experimented with other elution wavelengths of 214 nm, 222 nm, and 254 nm.

### 2.4 Characterization

The surface morphology of the R-ECNFs and A-ECNFs were analyzed using scanning electron microscopy (JOEL JSM-IT800 Schottky FESEM). The average fiber diameter was determined using image J software. The textural properties such as surface area and porosity of the fibers were investigated using N_2_ adsorption at 77 K by Brunauer–Emmett–Teller (Micromeritics ASAP 2060 BET Analyser). The chemical structures of the ECNFs were investigated using Fourier transform infra-red spectroscopy (Agilent ATR-FTIR) in the range 400–4,000 cm^−1^. The water contact angle measurement of the fibers was carried out using a Rame-Hart Goniometer/Tensiometer (Model 260-F4). The chemical composition of the fibers before and after adsorption was analyzed using Thermo Scientific Esclab Xi + XPS. The pH of solutions was measured using Thermo scientific Orion lab star pH meter.

## 3 Results and discussion

### 3.1 Characterization of the ECNFs

The ECNFs were characterized using different characterization techniques including SEM, BET, XPS, FTIR, and Goniometer to confirm the chemical, physical, structural, and morphological properties of the material.

#### 3.1.1 SEM imaging

**FIGURE 1:**
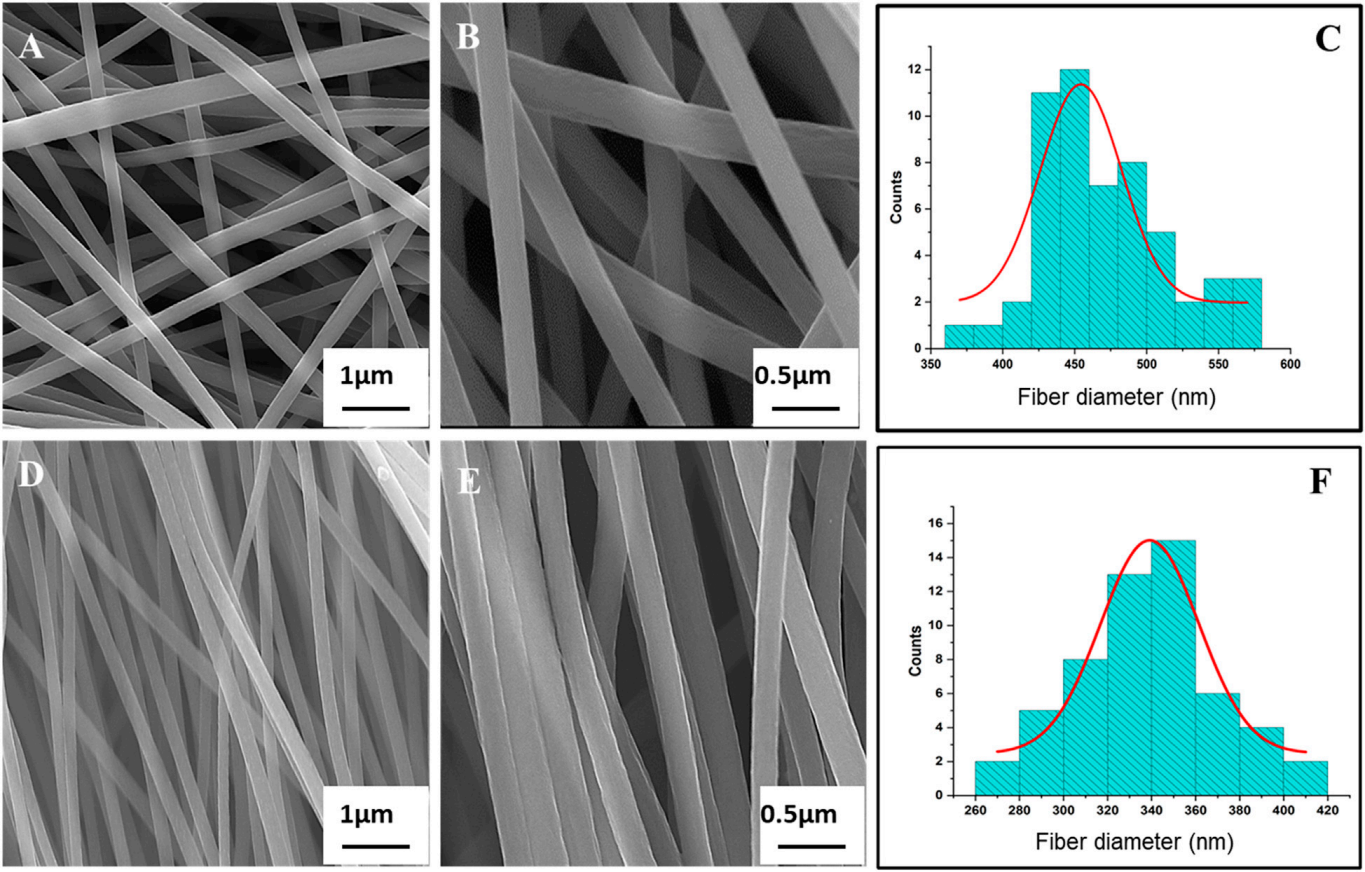
SEM images of **(A)** R-ECNFs low magnification and **(B)** R-ECNFs high magnification; **(D)** A-ECNFs low magnification and **(E)** A-ECNFs high magnification; Histogram distribution of fiber diameter (nm) for **(C)** R-ECNFs and **(F)** A-ECNFs.

Figures 1A, B, D, E presents both the SEM images of R-ECNFs and A-ECNFs. The R-ECNFs showed randomly distributed fibers with interconnected fiber spacing with macro-pores within the fibers, while the A-ECNFs exhibited fibers in a good unidirectional alignment with smooth surface and a uniform diameter after carbonization. The average fiber diameter is 454 ± 6 nm for R-ECNFs and 339 ± 2 nm for the A-ECNFs, respectively, according to the histogram distribution (Figures 1C, F).

#### 3.1.2 BET studies

**FIGURE 2:**
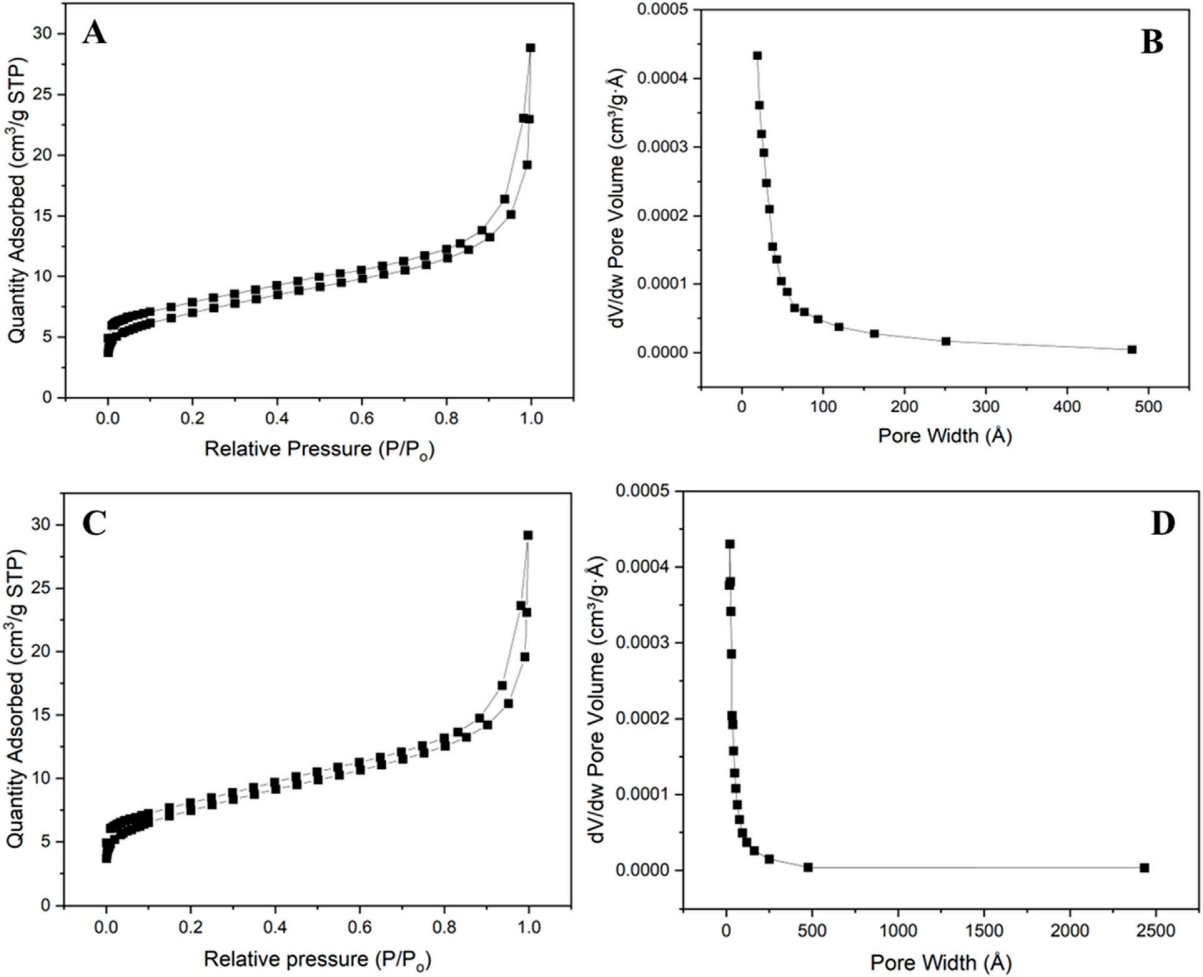
N_2_ adsorption-desorption isotherm for **(A)** R-ECNFs **(C)** A-ECNFS; pore size distribution for **(B)** R-ECNFs **(D)** A-ECNFs.

BET uses the adsorption of molecular nitrogen to calculate the surface area of a material (Gangupomu, Sattler, and Ramirez, 2016). The N_2_ gas adsorption and desorption isotherm was used to characterize the specific surface area, pore volume, and pore size distribution of ECNFs. The prepared ECNFs were degassed at 300°C for 4 h before the analysis. The BET surface area of both ECNFs were found to be between 20 and 26 m^2^/g (Table 1; Figure 2). The slight difference in the surface area in both fibers can be attributed to their structural orientation and possibly difference in the fibers’ diameter. This alignment allows for a more compact and orderly arrangement of the nanofibers. This organized structure may result in a negligible difference in BET surface area because of the accessibility of the nanofiber surfaces for gas adsorption. The R-ECNFs on the other hand are randomly oriented and therefore lack specific alignment pattern. This less organized arrangement may contribute to a slightly lower BET surface area compared to the A-ECNFs (Chen et al., 2021). However, the slight difference in the BET of the two fibers is not significant due to similar surface chemistry and morphology of both fibers.

**TABLE 1:** BET surface area and pore structure of the ECNFs.

Sample	Surface area (m^2^ g^−1^)	Total pore volume (cm^3^ g^−1^)	Micropore volume (cm^3^ g^−1^)	Mesopore volume (cm^3^ g^−1^)	Macropore volume (cm^3^ g^−1^)	Average pore size (nm)
R-ECNFs	24.46	0.025	0.001	0.006	0.017	6.78
A-ECNFs	26.29	0.030	0.001	0.007	0.022	7.65

#### 3.1.3 Wettability

The water contact angle of the respective fibers was measured as shown in Supplementary Figure S2. The R-ECNF exhibited hydrophobicity with an average water contact angle of 63° ± 4.3° while the A-ECNF has a more hydrophilic water contact angle of 10° ± 3.5°. The significant difference in the contact angles of the fibers explains the variation in their wettability. CNFs are naturally hydrophobic without functionalization (Cuervo et al., 2008; Mantripragada et al., 2023; Liu and Lu, 2006). High carbonization temperatures result in high graphitization, which increases their hydrophobicity (Li et al., 2020). Though both fibers were carbonized at the same temperature, the uniform directional spinning of the aligned fibers may contribute to the hydrophilicity of the A-CNFs. The alignment of the A-CNFs creates a more uniform and smoother surface which resulted in reduced surface roughness and lower contact angle. Additionally, the decrease in contact angle is resultant from water droplet spreading more easily down the nanofiber’s axis, without any obstacles hindering the movement of the contact line (Chen et al., 2021). Contrarily, the randomness of the R-CNFs resulted in more surface roughness which leads to higher contact angle (Bai et al., 2023).

#### 3.1.4 XPS and FTIR studies

**FIGURE 3:**
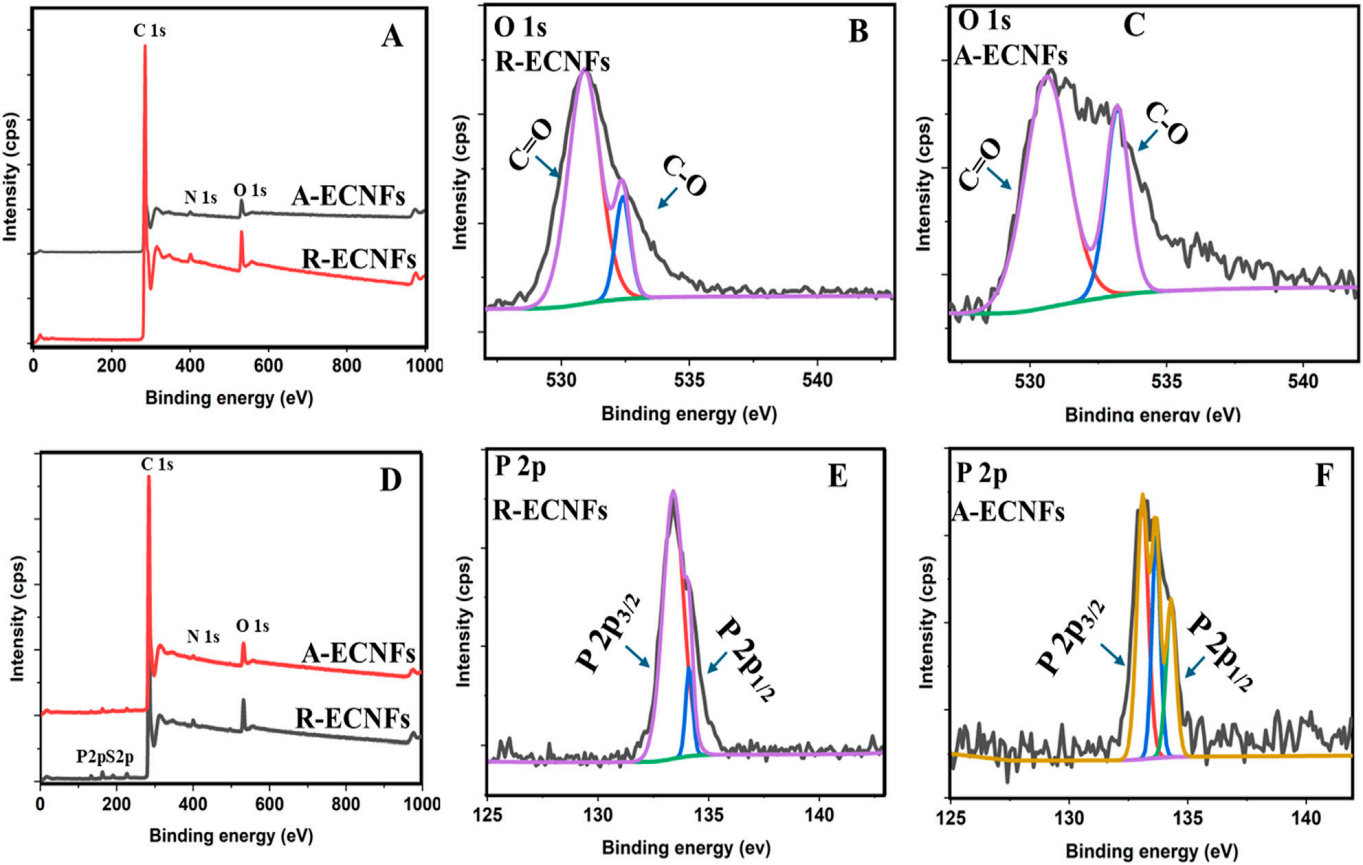
**(A)** Full XPS spectra of R-ECNFs and A-ECNFs, **(B)** High resolution elemental scan of O1s for R-ECNFs, and **(C)** O1s for A-ECNFs before OPP adsorption; **(D)** Full XPS spectra of R-ECNFs and A-ECNFs after OPP adsorption, **(E)** High resolution elemental scan of P2p for R-ECNFs **(F)** XPS spectrum of P2p for A-ECNF after OPP adsorption.

PAN precursor fibers undergo oxidative stabilization in air, and this process is greatly influenced by oxygen molecule diffusion (Zhou et al., 2010). The chemical composition of the ECNFs as well as the adsorbed ECNFs were further investigated using XPS. Both fibers exhibited similar chemical composition because similar peaks were displayed on the spectra. The XPS spectra of both ECNFs illustrate the presence of carbon (C1s), oxygen (O1s), and Nitrogen (N1s) by their distinctive peaks around 284 eV, 531 eV and 401 eV, respectively (Figure 3A). Carbon layers on the plane are created in carbon nano fibers when carbon atoms hybridize to create sp2 hybrid orbitals and are joined by covalent bonds to form a hexagonal ring. The weak Van der Waals interaction between these carbon layers is caused by non-localized π orbitals (Chen et al., 2020). The high-resolution spectra for carbon revealed peaks at 284.3 and 284.4 for R-ECNFs and A-ECNFs, respectively. Typically, C-O and C=O peaks in carbon spectra are reflected in single peak (Supplementary Figure S3). The π- π* satellite peak around 292 eV was also noticed in the high-resolution C1s spectra (Ashraf et al., 2013). The presence of nitrogen is thought to be caused by C-N bonds, which are normally present in PAN carbon fibers when the heat treatment’s final temperature is lower than 2,000°C (Zussman et al., 2005). The deconvolution of O 1s in both spectra presents peaks at the O 1s core level with peaks at ∼530.9 and ∼532.4 eV for R-ECNFs and 530.6 eV and 533.2 eV for A-ECNFs, respectively, corresponding to C=O, and C−O bonds (Figures 3B,C). Further analysis of high-resolution O 1s scan shows the ratio of the chemical bonds C=O to C-O is 82.8%–17.2% in the R-ECNFs while for A-ECNFs is 69.9% and 30.1%.

**FIGURE 4:**
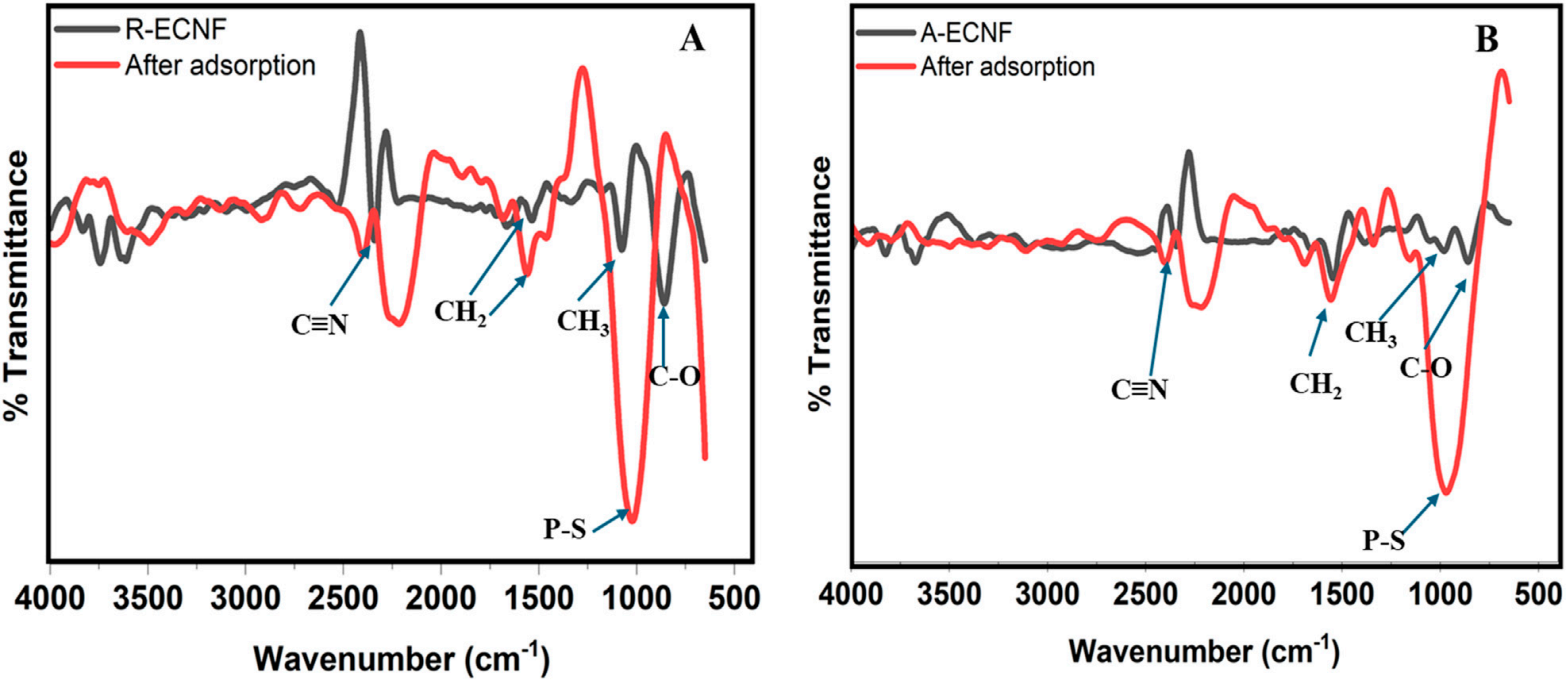
FTIR spectra of ECNFs of **(A)** R-ECNFs before and after adsorption, and **(B)** A-ECNF before and after adsorption.

The FTIR of both R-ECNFs and A-ECNFs showed similar vibrations owing to the similar functional groups present on their surface. This is in good agreement with the XPS data. As shown in Figure 4, the FTIR spectra of R-ECNFs exhibited characteristic peak at 2,217 cm^−1^, arising from the C≡N nitrile group vibration (Ibupoto et al., 2018; Maddah et al., 2017). Vibrations at 1,569 cm^−1^, 1,447 cm^−1^ and 1,015 cm^−1^ correspond to the vibrations of the aliphatic C-H (CH, CH_2_ and CH_3_) and C-O bonds (Thamer et al., 2019). While the FTIR spectra of A-ECNFs were observed at 2,228 cm^−1^, 1,569 cm^−1^, 1,337 cm^−1^ and 965 cm^−1^ which are also suggestive of C≡N nitrile bond, aliphatic C-H bonds, and C-O bonds vibrations respectively. In terms of the phase compositions and crystallographic structures of ECNFs, we have done the X-ray diffraction (XRD) measurement of the same type of ECNFs made using the same protocol (Yin et al., 2021). The ECNFs mostly exist as graphitic-type lattice structures.

### 3.2 Adsorption studies

#### 3.2.1 XPS and FTIR of OPP adsorption

The XPS spectra of the ECNFs after OPP adsorption, however, showed introduction of the new peaks in the region of 133 eV and 163 eV (Figures 3E,F). These new peaks illustrate the presence of phosphate and sulphate bonds respectively in the adsorbed fibers. Further deconvolutions of the P 1s spectra revealed peaks at 133.4 eV and 134.12 eV for R-ECNF while the A-ECNF displayed peaks at 133.1, 133.67 and 134.3 eV, which correspond to Phosphate bond splitting. The results confirmed the binding of the organophosphate unto the surface of the fibers. In addition, it is important to highlight that the chemical bond percentages exhibit significant variation between the two types of fibers (Supplementary Table S1). Specifically, R-ECNF is characterized by a Phosphorus-Oxygen (P-O) chemical composition of P 2p splitting of percentage 89.9% 2P_3/2_ and 10.1% 2P_1/2_. In contrast, A-ECNF displays a more complex distribution of chemical bonds with multiple distinct chemical states due to variation in the electronic structure and bonding. For A-ECNF, the bond percentages are more varied displaying P-O chemical bond composition of the same of 46.8% 2P_3/2_, 29.0% 2P_3/2_ and 24.28% 2P_1/2_. The difference in the chemical bonding between both fibers suggest slightly different structural properties. The results indicate that the P-O bond is more presence at the R-ECNFs according to the P 2p intensity (∼2× of the A-ECNFs) which further justifies the better adsorption efficiency as indicated by the chemical bond intensity and atomic percentages from the XPS data (Figure 3; Supplementary Table S1).

Conventionally, the relationship between surface chemistry and adsorption properties is used to predict the mechanism of adsorption between CNF and organic molecule. FTIR was also used to study the structure and chemical bonds of the carbon nanofibers after adsorption. The pesticide FTIR spectra (Supplementary Figure S1) show characteristic peaks at 2,975 cm^−1^ and 2,926 cm^−1^ which are attributed to strong symmetric C-H bonds. Similar vibrations at 1,458 cm^−1^ and 1,386 cm^−1^ are also attributed to medium C-H bonds. The two vibrations around 793 cm^−1^ and 694 cm^−1^ are attributed to strong *P*=S and S-P=S, a good agreement with literature (Asan Mohamed and Janaki, 2021). Additionally, vibrations were observed at 1,015 cm^−1^ and 948 cm^−1^ which are suggestive of carbonyl bond (Asan Mohamed and Janaki, 2021; Yang et al., 2019). After adsorption, there are emergence of new peaks on both nanofibers in FTIR spectra (Figure 4). A reduction in the intensity of the carbonyl bond was observed on both fibers with a slight shift observed after OPP adsorption. The surface adsorption of the organophosphate was confirmed by the appearance of vibrations at 860 cm^−1^ and 854 cm^-1^ for R-ECNF and A-ECNF (Figure 4) which correspond to the shift in the P-S bond. It is worthy of note that the intensity of the P-S vibration on the adsorbed R-CNF is more than the A-ECNFs, suggesting a stronger adsorption on the surface of the R-ECNF. Moreover, the peaks at 1,447 cm^−1^ and 1,569 cm^−1^ shifted to 1,535 cm^−1^ and 1,668 cm^−1^ on the R-ECNF, respectively. This shift may be due to ethion adsorption by Vander-Waal forces on the surface and π-π interactions (Ibupoto et al., 2018). The π-π interactions may have played a significant role in the formation of the carbon conjugation within the system (the ECNFs and π acceptors of ethion) (Firozjaee et al., 2017). During carbonization, most of the oxygen and nitrogen atoms in the PAN macromolecular chain are removed, and the majority of the carbon atoms are connected to one another via sp2 hybridization to create substantial delocalized π bonds which aid the interaction with the organophosphate (Zhang et al., 2022).

The increased surface roughness and surface energy in the R-ECNFs improves hydrophobicity which enhances the adsorption. Research has shown that hydrophobic surfaces tend to show more affinity to hydrophobic substances such as oil and OPPs (Zhang et al., 2022). Ethion is a hydrophobic pesticide (Log K_ow_ = 5.073) (ATSDR, 2000) therefore, it is assumed that hydrophobic interaction is most likely between the R-ECNFs and ethion. Despite the relatively lower surface areas, the R-ECNFs showed good adsorption capacity for organophosphate pesticides without further functionalization.

#### 3.2.2 Effect of adsorbent dosage

**FIGURE 5:**
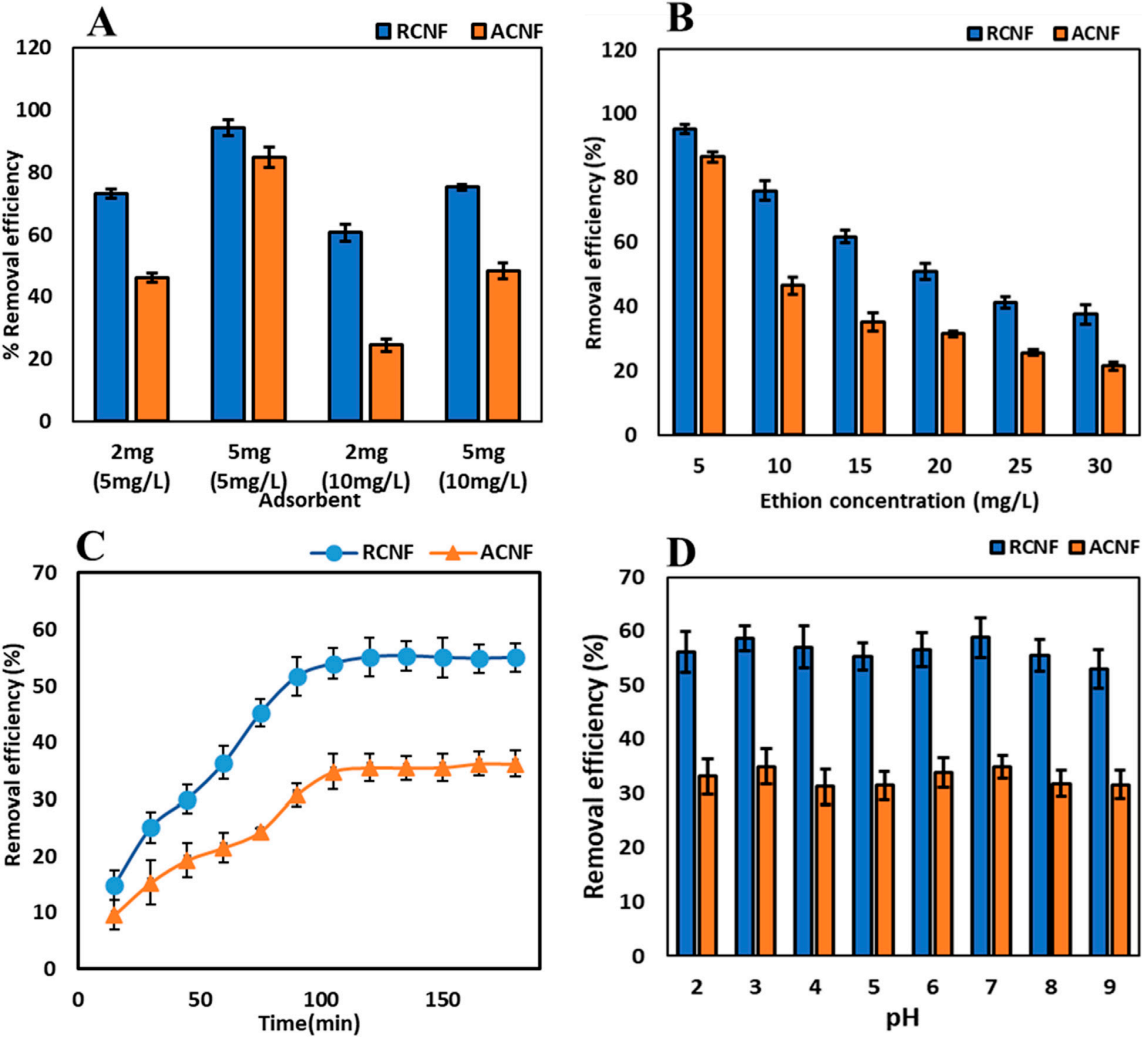
Ethion adsorption results: **(A)** effect of dosage, **(B)** effect of pesticides concentration, **(C)** Effect of time, and **(D)** effect of pH on removal efficiency.

A crucial factor in determining the quantitative removal of the chosen analyte is the dosage of the adsorbent. The effect of the amount of ECNFs on the adsorption efficiency was investigated using two adsorbent dosages 2 mg and 5 mg for the adsorption study from 10 mL of two different ethion concentrations (5 mg/L and 10 mg/L). Figure 5A shows the removal efficiency increased with 5 mg dosage compared to 2 mg. This is attributed to increase in the available unsaturated binding sites that are available on the carbon nanofiber as a result of increased dosage (Ibupoto et al., 2018; Firozjaee et al., 2017). This means that more ethion molecules can interact with those adsorption sites and enhance the adsorption of the pesticide. Based on these results and better accuracy in this study, 5 mg of the ECNFs were used for further adsorption studies.

#### 3.2.3 Effect of OPP concentration

The effect of increasing ethion concentration on the adsorption capacity was studied at concentrations ranging from 5 mg/L to 30 mg/L using both ECNFs. Under similar conditions, the efficiency of the R-ECNFs exceeds that of the A-ECNFs. While 5 mg/L has the highest removal efficiency for both fibers, the removal efficiency decreases with increasing ethion concentration as shown in Figure 5B. This is attributed to the limited active sites on the surface of the CNF which resulted in decrease in adsorption efficiency. Contrarily, the adsorption capacity increases for 5 mg/L concentration till it reaches about 20 mg/L concentration for both R-ECNFs and A-ECNFs. The R-ECNFs have a greater adsorption capacity of 20 mg/g than A-ECNFs which have adsorption capacity of 12 mg/g. This may be attributed to the hydrophobic and π-π interactions between the surface functional groups of the fibers and the pesticide. The higher concentration of the OPP in solution increases the adsorption capacity until the saturation of the binding sites (Thamer et al., 2019).

#### 3.2.4 Effect on time and pH

The removal of ethion was carried out at different time interval ranging from 0 to 180 min to determine the optimum time required for maximum adsorption capacity. The 20 mg/L ethion was used for the kinetics experiment while keeping the neutral pH and the dosage constant. It was observed that there was a progressive increase in the removal efficiency until above 100 min as shown in Figure 5C. Maximum percentage removal was observed at about 120 min which was used for the adsorption process. This implies it needs 120 min for the vacant sites on the surface of the CNF to reach ethion saturation (Firozjaee et al., 2017).

Studies have shown that the pH of the adsorbate solution significantly influences the adsorption process as shown in Figure 5D. The effect of pH on the adsorption of ethion was studied from 2 to 9 using 20 mg/L concentration, 5 mg of adsorbent for 120 min. It was discovered that there was no substantial variance in the efficiency of removal observed in the range pH 2-9, which suggests a relative stability. The results imply the adsorption mechanism of π-π interaction of the carbon fiber with the OPP, because the protonation and deprotonation at different pH had insignificant impact on ethion adsorption (Maddah et al., 2017).

#### 3.2.5 Adsorption isotherm

An adsorption isotherm describes the equilibrium relationship between the adsorbate present in the solution and the adsorbate that has been adsorbed onto the surface of the adsorbent. In this research, Langmuir and Freundlich isotherm models were fitted into the ethion adsorption on ECNFs. Langmuir isotherm assumes the monolayer adsorption of molecules onto the surface of the adsorbent with a limited number of homogeneous sites. The model is represented with the following Equation 4 (Ibupoto et al., 2018).

$$q_{e}=\frac{K_{L}C_{e}q_{m}}{1+K_{L}C_{e}}$$ (4)

where *C*_*e*_ is the equilibrium concentration of the pesticide in solution (mg/L), *q*_*e*_ is the amount of pesticide adsorbed per unit mass of the adsorbent, *q*_*m*_ is the maximum adsorption capacity and K_L_ is Langmuir constant.

Freundlich isotherm assumes a multilayer adsorption on heterogeneous surfaces, and it is described by Equation 5:

$$q_{e}=K_{f}C_{e}^{\frac{1}{n}}$$ (5)

where *q*_e_ is the amount of the solute adsorbed per unit mass of the adsorbent, *C*_*e*_ is the equilibrium concentration of the adsorbate, *K*_*f*_ is the Freundlich constant, 1/n is the heterogeneity factor or a constant that predicts the adsorption strength. When 1/n is between 0 and 1, the adsorption is favorable (Ibupoto et al., 2018).

**FIGURE 6:**
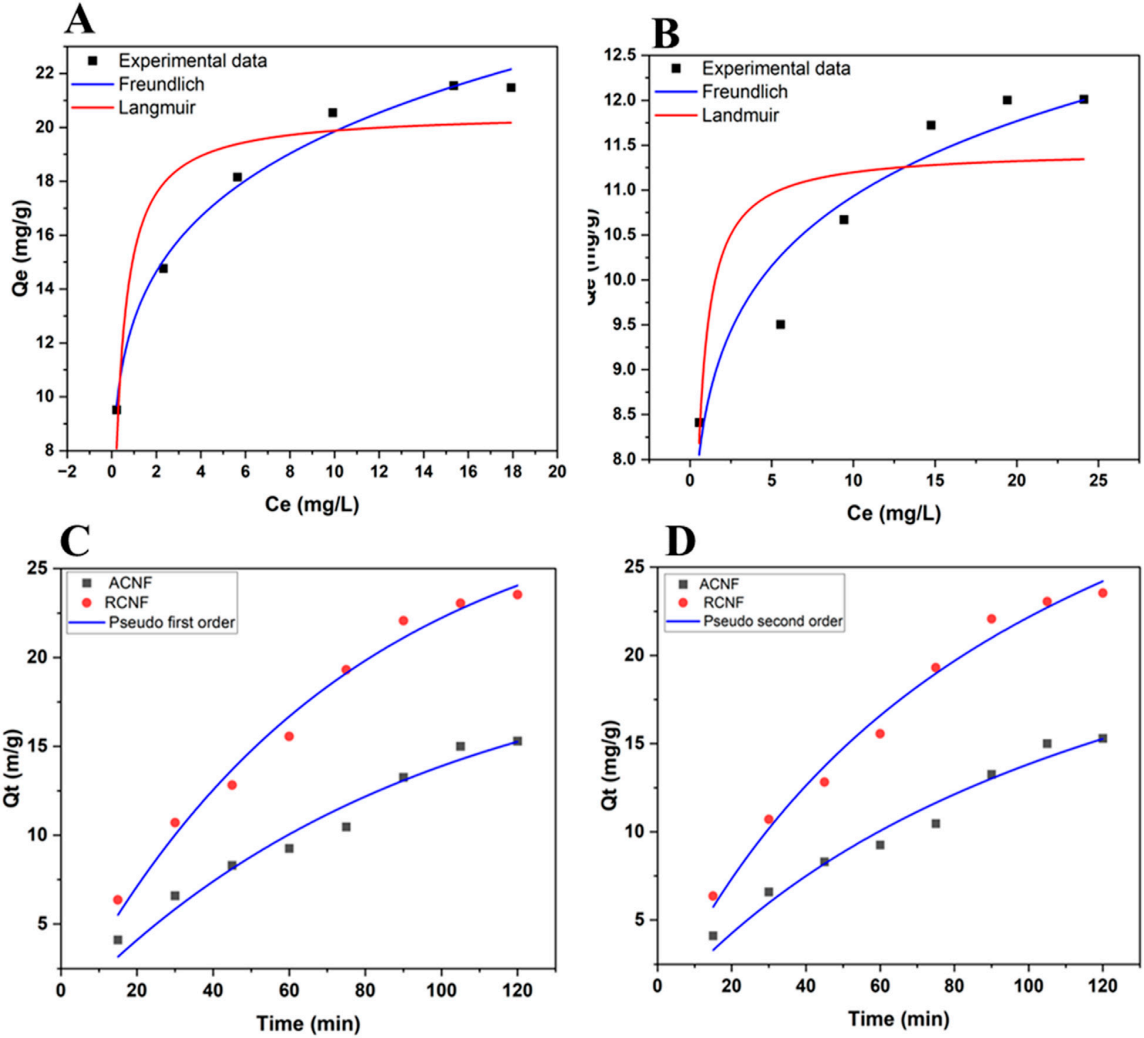
Langmuir and Freundlich adsorption isotherm of ethion adsorption fitting at **(A)** R-ECNFs and **(B)** A-ECNFs; The adsorption kinetics fitting of **(C)** Pseudo first order and **(D)** Pseudo second order plots for R-ECNFs and A-ECNFs.

The experimentally determined adsorption isotherms and their parameter constants using nonlinear fitting are shown in Table 2. Figure 6 shows the fitting plots of the two models. Based on the correlation coefficient *R* ^2^ value of the two adsorption isotherms, the adsorption data fits the Freundlich adsorption isotherm model better than the Langmuir model. The result suggests multilayer ethion adsorption at the surfaces of the fibers. Table 2 also shows the heterogeneity factor, 1/n values are less than one which is an indication that the adsorption process is favorable.

**TABLE 2:** Adsorption isotherm parameters of both fibers.

	Langmuir isotherm			Freundlich isotherm		
	Qmax (mg/g)	K_L_ (L/mg)	*R* ^2^	1/n	K_F_(L/g)	*R* ^2^
RCNF	20.56 ± 1.15	2.91 ± 1.29	0.843	0.18	12.84 ± 0.36	0.989
ACNF	11.79 ± 0.61	3.92 ± 2.11	0.670	0.12	8.50 ± 0.58	0.916

#### 3.2.6 Adsorption kinetics

Adsorption kinetics plays a crucial role in explaining the nature of the transitory behavior of the solute as it moves from solution to the surface of the adsorbent. For this study, two kinetic models, namely, pseudo first-order (PFO) and pseudo-second order (PSO) were used to describe the mechanism of the organophosphate adsorption on the CNFs. The PFO and PSO rate are depicted as Equations 6, 7 (Musah et al., 2022):

$$\frac{dq_{t}}{\text{dt}}=k_{1}\left(q_{e}-q_{t}\right)$$ (6)

Pseudo first order kinetic model

$$\frac{dq_{t}}{dt}=k_{2}{\left(q_{e}-q_{t}\right)}^{2}$$ (7)

Pseudo second order kinetic model.

where q_e_ (mg/g) is the amount of pesticide adsorbed at equilibrium, q_t_ (mg/g) is the amount of pesticide adsorbed at time t (min), k_1_ (min^−1^) is the rate constant of the PFO model and k_2_ (min^−1^) is the rate constant of the PSO model.

The adsorption kinetics parameters and correlation coefficients are displayed in Table 3. The *R* ^2^ values of both kinetic models are close to each other which is an indication that the adsorption process could be well described by PFO and PSO. However, *R* ^2^ value of the PFO kinetic model is slightly higher than that of PSO which is a good indication that ethion adsorption follows PFO kinetic model better. This indicates that the adsorption process is influenced by the properties and interactions of both the adsorbate and the adsorbent. Since the multilayer adsorbate form during the adsorption process showing a better fit to Freundlich isotherm model, the kinetics of first ethion layer onto the surface of fiber is different from the adsorption of addition layer. It is plausible that it can be explained by a non-integer-order kinetics of the ethion adsorption (Skopp, 2009). Similar results are reported for organophosphate pesticides using carbon nanotube-based absorbent (Firozjaee et al., 2017; Dehghani et al., 2021).

**TABLE 3:** Adsorption kinetic parameters of both fibers.

	Pseudo first order			Pseudo second order		
	K_1_ (min^−1^)	Q_e_ (mg g^−1^)	*R* ^2^	K_2_ (min^−1^) (E)	Qe (mg g^−1^)	*R* ^2^
R-ECNF	0.013 ± 0.002	29.89 ± 2.23	0.984	2.193 E±6.29–5	44.751 ± 4.59	0.984
A-ECNF	0.011 ± 0.003	20.88 ± 3.26	0.966	2.428 E±1.18–4	31.77 ± 5.88	0.963

### 3.3 Proposed adsorption mechanism

Considering the inherently hydrophobic nature of organic pesticides, the primary sorption mechanisms are believed to involve hydrophobic interactions, Vander Waal interactions and π interactions. π-π interaction is also generally considered to be a significant factor enhancing the adsorption of aromatic compounds on carbon materials. Further insight into the adsorption process is gleaned from the evaluation of XPS and FTIR analysis. The FTIR data shows shift in the C=C vibration from 1,569 cm^−1^ to 1,535 cm^−1^ in the R-ECNF suggests significant changes in the surface chemistry and bonding configurations. Similar shift is also observed in the A-ECNF. The reduction in the carbonyl vibrations on the adsorbed fibers also suggest the transition of the bonding orbital of the pesticide molecule. This shift in chemical structure suggests π-π stacking between the carbon nano fibers and the ethion molecule. Carbon nanofibers are composed of sp2 hybridized carbon atoms which gives the conjugated polycyclic structure. The supramolecular forces in the carbon nanostructure and the C-C π stacking were assumed to play a significant role in the adsorption process. Furthermore, it is largely suspected that hydrophobic interactions-which is believed to be enhanced by the hydrophobicity of the pesticide’s molecules-also contributed considerably to the adsorption process. In addition, Vander Waal forces also contribute to the adsorption of the ethion molecule on the surface of the fibers as this forms one of the predominant mechanisms of sorption for non-functionalized carbon-based materials (Uddin, 2021).

### 3.4 Comparison of OPP adsorption with other adsorbent in the literature

Table 4 provides a summary of relevant experimental data, highlighting the results of batch experiments conducted with various adsorbents and under different operating conditions for the adsorption of organophosphate pesticides from aqueous solutions. The table includes a detailed comparison of adsorption removal data across a broad spectrum of operating parameters used in these adsorption studies. The adsorption data for (E-CNFs) presented in this study aligns well with the findings reported in the existing literature, indicating the suitability of E-CNFS in the removal of organophosphate pesticides.

**TABLE 4:** Comparison of some experimental data for adsorption of OPPs from aqueous solution using different materials.

OPP	Adsorbent	Adsorbent dosage	OPP concentration	Time	Adsorption efficiency (%)	Adsorption capacity (mg/g)	Ref
Diazinon	Nanocrystalline MgO	0.05–0.10 g	0.30 g/L	24 h	—	20	Armaghan and Amini, (2017)
Ethion	Cu-BTC	112.5 mg	20 mg/L	180 mins	97	182	Abdelhameed et al. (2016)
Dimethoate	Gold nanorods	2–200 mg/L	2–1,150 mg/L	24 h	—	57	Momić et al. (2016)
Malathion	MOF	5 mg	1 mg/L	60 min	91	14	Sagar and Kukkar, (2023)
Diazinon	Advanced Coconut Shell biochar	5 g/L	1 mg/L	120 min	98	10.33	Baharum et al. (2020)
Malathion	MWCNT	0.1–0.3 g/L	6 mg and 10 mg	30 min	100	—	Dehghani et al. (2017)
Ethion	E-CNFs	5 mg	5–20 mg	120 min	96	20	This study

### 3.5 Reusability and real-world application

**FIGURE 7:**
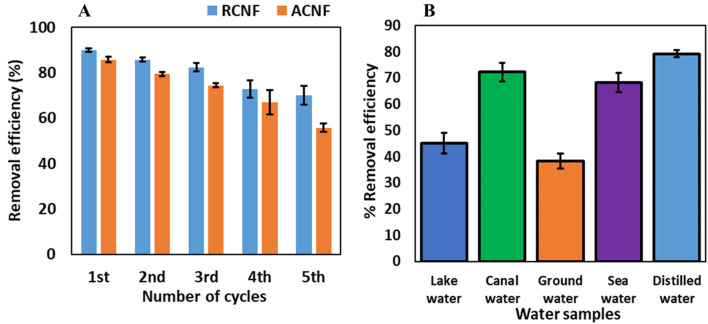
**(A)** Recyclability result of R-ECNFs and A-ECNFs, and **(B)** adsorption results of different water samples.

The potential for an adsorbent to be reused enhances its value and cost-effectiveness in adsorption processes. To investigate the reusability of the CNF, the R-ECNFs were desorbed using methanol. The CNFs were soaked in methanol for about 2 h to ensure complete desorption of the adsorbed pesticides, then washed thrice with distilled water and dried for reuse. Adsorption experiments using 5 mg dosage of ECNFs, 10 mL 5 mg/L of ethion were subsequently carried out for five cycles of 120 min. The R-ECNFs exhibited removal efficiencies between 80% and 90% until the third cycle, gradually declining to approximately 70% removal efficiency for the fourth and fifth cycles, as illustrated in Figure 7A. This trend underscores the practical and economic viability of the material, demonstrating its potential for multiple reuses.

To assess the real-world applicability of the material, R-ECNFs were employed as an adsorbent to remove ethion from artificially spiked water samples collected from various sources, including Lake Okeechobee, EREC canal, EREC groundwater, and seawater from Lake Worth (all sourced from Clemson University, South Carolina, United States). Prior to spiking, the presence or absence of ethion in the water samples was determined through analysis using HPLC. Subsequently, the water samples were spiked with 10 mg/L ethion and tested to calculate the percentage recovery which is the measure of analyte recovered from the spiked solution. The 10 mg/L spike concentration was employed to replicate real world sample concentration prediction. The achieved percentage recovery values exceeded 85%, falling within the range of 70%–130% stipulated by the United States Environmental Protection Agency (EPA) (Wanjeri et al., 2018). Under optimized conditions, the R-ECNF was utilized to adsorb ethion from the spiked water samples and the concentrations were determined using HPLC-DAD. The results, illustrated in Figure 7B, exhibited removal efficiencies of 45%, 72%, 38%, and 68% for Lake Okeechobee, EREC canal, EREC groundwater, and seawater from Lake Worth, respectively. Despite the complex matrix of the water samples, these outcomes further validate the material’s suitability for removing ethion from different water sources in practical real-world applications.

## 4 Conclusion

Electrospun carbon nanofibers (ECNFs) were used as a highly effective nano-adsorbent in the removal of organophosphate pesticide ethion. The fabrication of these CNFs involved the electrospinning method, followed by subsequent stages of stabilization and carbonization. The research findings demonstrated robust adsorption efficiency for the random and aligned ECNFs. Notably, the R-ECNFs exhibited a superior efficiency in comparison to the A-ECNFs, showcasing their improved efficacy in the removal process. Additionally, isotherm studies indicated a multilayer adsorption pattern, fitting better into the Freundlich isotherm model. Furthermore, the kinetics data corresponded a non-integer-order kinetics model, providing substantial evidence for the physical adsorption nature of the process. In-depth analysis using XPS and FTIR conducted before and after the adsorption process offered valuable insights into the adsorption mechanism involving the π-π stacking of ethion on carbon fiber, hydrophobic and Vander Waal forces. The fiber material stability and applicability to real world samples were confirmed through the recyclability test giving good reusability results beyond three times. In summary, this comparative study gave better understanding of the morphology and surface functionality of the fibers regarding the influences on the adsorption process. The material offers good suitability for organophosphate removal in water offering a promising solution for efficient and sustainable wastewater treatment solutions.

## Data Availability

The original contributions presented in the study are included in the article/Supplementary Material, further inquiries can be directed to the corresponding authors.

## References

[B1] Abdelhameed R. M. Abdel-Gawad H. Elshahat M. Emam H. E. (2016). Cu-BTC@cotton composite: design and removal of ethion insecticide from water. RSC. Adv. 6 (48), 42324–42333. 10.1039/c6ra04719j

[B2] Allado K. Liu M. Jayapalan A. Arvapalli D. Nowlin K. Jianjun W. (2021). Binary MnO2/Co3O4metal oxides wrapped on superaligned electrospun carbon nanofibers as binder free supercapacitor electrodes. Energy Fuels 35 (9), 8396–8405. 10.1021/acs.energyfuels.1c00556

[B3] Alrefaee S. H. Aljohani M. Alkhamis K. Shaaban F. El-Desouky M. G. El-Bindary A. A. (2023). Adsorption and effective removal of organophosphorus pesticides from aqueous solution via novel metal-organic framework: adsorption isotherms, kinetics, and optimization via Box-Behnken design. J. Mol. Liq. 384, 122206. 10.1016/j.molliq.2023.122206

[B4] Armaghan M. Amini M. M. (2017). Adsorption of diazinon and fenitrothion on nanocrystalline magnesium oxides. Arabian J. Chem. 10 (1), 91–99. 10.1016/j.arabjc.2014.01.002

[B5] Asan Mohamed B. Janaki P. (2021). Determination of active ingredients in commercial insecticides using spectral characteristics of Fourier transform infrared spectroscopy (FTIR). J. Appl. Nat. Sci. 13 (SI), 110–123. 10.31018/jans.v13iSI.2809

[B6] Ashraf A. Dastgheib S. A. Mensing G. Shannon M. A. (2013). Surface characteristics of selected carbon materials exposed to supercritical water. J. Supercrit. Fluids 76, 32–40. 10.1016/j.supflu.2013.01.017

[B7] ATSDR (2000) Toxicological profile for ethion. (Atlanta, GA: U.S. Department of Health and Human Services, Public Health Service). Available at: https://www.atsdr.cdc.gov/ToxProfiles/tp152.pdf.

[B8] Awad R. Mamaghani A. H. Boluk Y. Hashisho Z. (2021). Synthesis and characterization of electrospun PAN-based activated carbon nanofibers reinforced with cellulose nanocrystals for adsorption of VOCs. Chem. Eng. J. 410, 128412. 10.1016/j.cej.2021.128412

[B9] Baharum N. A. Nasir H. M. Ishak M. Y. Isa N. M. Ali Hassan M. Aris A. Z. (2020). Highly efficient removal of diazinon pesticide from aqueous solutions by using coconut shell-modified biochar. Arabian J. Chem. 13 (7), 6106–6121. 10.1016/j.arabjc.2020.05.011

[B10] Bai L. Wang X. Sun X. Jiao L Huang L. Sun H. (2023). Enhanced superhydrophobicity of electrospun carbon nanofiber membranes by hydrothermal growth of ZnO nanorods for oil–water separation. Arabian J. Chem. 16 (3), 104523. 10.1016/j.arabjc.2022.104523

[B11] Bhandari G. Atreya K. Scheepers P. T. J. Geissen V. (2020). Concentration and distribution of pesticide residues in soil: non-dietary human health risk assessment. Chemosphere 253, 126594. 10.1016/j.chemosphere.2020.126594 32289601

[B12] Chawla P. Kaushik R. Shiva Swaraj V. J. Kumar N. (2018) Organophosphorus pesticides residues in food and their colorimetric detection. Environ. Nanotechnol. Monit. Manag. 10 (3), 292–307. 10.1016/j.enmm.2018.07.013

[B13] Chen C. H. Dai L.L Chuang A. D. C. Dash B. S. Chen J. P. (2021). Tension stimulation of tenocytes in aligned hyaluronic acid/platelet-rich plasma-polycaprolactone core-sheath nanofiber membrane scaffold for tendon tissue engineering. Int. J. Mol. Sci. 22 (20), 11215. 10.3390/ijms222011215 34681872 PMC8537129

[B14] Chen X. Wang X. Fang D. (2020) A review on C1s XPS-spectra for some kinds of carbon materials. Fuller Nanotube. 28 (2), 1048–1058. 10.1080/1536383X.2020.1794851

[B15] Cuervo M. R. Asedegbega-Nieto E. Díaz E. Vega A. Ordóñez S. Castillejos-López E. (2008). Effect of carbon nanofiber functionalization on the adsorption properties of volatile organic compounds. J. Chromatogr. A 1188 (2), 264–273. 10.1016/j.chroma.2008.02.061 18325528

[B16] Degrendele C. Klánová J. Prokeš R. Příbylová P. Šenk P. Šudoma M. (2022). Current use pesticides in soil and air from two agricultural sites in South Africa: implications for environmental fate and human exposure. Sci. Total. Environ. 807 (Pt 1), 150455. 10.1016/j.scitotenv.2021.150455 34634720

[B17] Dehghani M. H. Hassani A. H. Rao Karri R. Younesi B. Shayeghi M. Salari M. (2021). Process optimization and enhancement of pesticide adsorption by porous adsorbents by regression analysis and parametric modelling. Sci. Rep. 11 (1), 11719. 10.1038/s41598-021-91178-3 34083608 PMC8175395

[B18] Dehghani M. H. Kamalian S. Shayeghi M. Yousefi M. Heidarinejad Z. Agarwal S. (2019). High-performance removal of diazinon pesticide from water using multi-walled carbon nanotubes. Microchem. J. 145, 486–491. 10.1016/j.microc.2018.10.053

[B19] Dehghani M. H. Niasar Z. S. Reza Mehrnia M. Shayeghi M. Al-Ghouti M. A. Heibati B. (2017). Optimizing the removal of organophosphorus pesticide malathion from water using multi-walled carbon nanotubes. Chem. Eng. J. 310, 22–32. 10.1016/j.cej.2016.10.057

[B20] Derbalah A. Chidya R. Jadoon W. Sakugawa H. (2019). Temporal trends in organophosphorus pesticides use and concentrations in river water in Japan, and risk assessment. J. Environ. Sci. China 79, 135–152. 10.1016/j.jes.2018.11.019 30784439

[B21] Dissanayake N. M. Arachchilage J. S. Samuels T. A. Obare S. O. (2019). Highly sensitive plasmonic metal nanoparticle-based sensors for the detection of organophosphorus pesticides. Talanta 200, 218–227. 10.1016/j.talanta.2019.03.042 31036176

[B22] Firozjaee T. T. Mehrdadi N. Baghdadi M. Nabi Nabi Bidhendi G. R. (2017). The removal of diazinon from aqueous solution by chitosan/carbon nanotube adsorbent. Desalination. Water. Treat. 79, 291–300. 10.5004/dwt.2017.20794

[B23] Gangupomu R. H. Sattler M. L. Ramirez D. (2016). Comparative study of carbon nanotubes and granular activated carbon: physicochemical properties and adsorption capacities. J. Hazard. Mater. 302, 362–374. 10.1016/j.jhazmat.2015.09.002 26476807

[B24] Gui X. Hu J. Han Y. (2019). Random and aligned electrospun gelatin nanofiber mats for human mesenchymal stem cells. Mater. Res. Innovations 23 (4), 208–215. 10.1080/14328917.2018.1428073

[B25] Huang C.Y Hu K. H. Zung H. W. (2016). Comparison of cell behavior on pva/pva-gelatin electrospun nanofibers with random and aligned configuration. Sci. Rep. 6, 37960. 10.1038/srep37960 27917883 PMC5137148

[B26] Ibupoto A. S. Qureshi U. A. Ahmed F. Khatri Z. Khatri M. Maqsood M. (2018). Reusable carbon nanofibers for efficient removal of methylene blue from aqueous solution. Chem. Eng. Res. Des. 136, 744–752. 10.1016/j.cherd.2018.06.035

[B27] Kaushal J. Khatri M. Arya S. K. (2021). A treatise on organophosphate pesticide pollution: current strategies and advancements in their environmental degradation and elimination. Ecotoxicol. Environ. Saf. 207, 111483. 10.1016/j.ecoenv.2020.111483 33120277

[B28] Kim J.In Tae In H. Erik Aguilar L. Chan H. P. Kim C. S. (2016). A controlled design of aligned and random nanofibers for 3D Bi-functionalized nerve conduits fabricated via a novel electrospinning set-up. Sci. Rep. 6, 23761. 10.1038/srep23761 27021221 PMC4810461

[B29] Li J. Zhang W. Zhang X Huo L. Liang J. Wu L. (2020). Copolymer derived micro/meso-porous carbon nanofibers with vacancy-type defects for high-performance supercapacitors. J. Mater. Chem. A 8 (5), 2463–2471. 10.1039/c9ta08850d

[B30] Liu Y. Zeng Z. Bloom B. Waldeck D. H. Jianjun W. (2018). Stable low-current electrodeposition of α-MnO2 on superaligned electrospun carbon nanofibers for high-performance energy storage. Small 14 (3). 10.1002/smll.201703237 29193670

[B31] Liu Y. C. Lu Da N. (2006). Surface energy and wettability of plasma-treated polyacrylonitrile fibers. Plasma Chem. Plasma. Process. 26 (2), 119–126. 10.1007/s11090-006-9005-7

[B32] Maddah B. Soltaninezhad M. Adib K. Hasanzadeh M. (2017). Activated carbon nanofiber produced from electrospun PAN nanofiber as a solid phase extraction sorbent for the preconcentration of organophosphorus pesticides. Sep. Sci. Technol. Phila. 52 (4), 700–711. 10.1080/01496395.2016.1221432

[B33] Mahar F. K. He L. Wei K. Mehdi M. Zhu M. Gu J. (2019). Rapid adsorption of lead ions using porous carbon nanofibers. Chemosphere 225, 360–367. 10.1016/j.chemosphere.2019.02.131 30884297

[B34] Mahpishanian S. Sereshti H. Baghdadi M. (2015). Superparamagnetic core-shells anchored onto graphene oxide grafted with phenylethyl amine as a nano-adsorbent for extraction and enrichment of organophosphorus pesticides from fruit, vegetable and water samples. J. Chromatogr. A 1406, 48–58. 10.1016/j.chroma.2015.06.025 26129984

[B35] Mali H. Shah C. Raghunandan B. H. Prajapati A. S. Patel D. H. Trivedi U. (2022). Organophosphate pesticides an emerging environmental contaminant: pollution, toxicity, bioremediation progress, and remaining challenges. J. Environ. Sci. 127, 234–250. 10.1016/j.jes.2022.04.023 36522056

[B36] Mantripragada S. Deng D. Zhang L. (2023). Algae-enhanced electrospun polyacrylonitrile nanofibrous membrane for high-performance short-chain PFAS remediation from water. Nanomaterials 13 (19), 2646. 10.3390/nano13192646 37836287 PMC10574606

[B37] Mantripragada S. Gbewonyo S. Deng D. Zhang L. (2020). Oil absorption capability of electrospun carbon nanofibrous membranes having porous and hollow nanostructures. Mater. Lett. 262, 127069. 10.1016/j.matlet.2019.127069

[B38] Mehta J. Kumar Dhaka R. Dilbaghi N. Lim D. K. Ashraf A. H. Kim Ki H. (2022). Recent advancements in adsorptive removal of organophosphate pesticides from aqueous phase using nanomaterials. J. Nanostructure. Chem. 14, 53–70. 10.1007/s40097-022-00516-y

[B39] Mokhena T. C. Matabola K. P. Mokhothu T. H. Mtibe A. Mochane M. J. Ndlovu G. (2022). Electrospun carbon nanofibres: preparation, characterization and application for adsorption of pollutants from water and air. Sep. Purif. Technol. 288, 120666. 10.1016/j.seppur.2022.120666

[B40] Momić T. Tamara L. P. Una B. Vesna V. Ana M. Zlatko R. Vladimir B. P. Vesna V (2016). Adsorption of organophosphate pesticide dimethoate on gold nanospheres and nanorods. J. Nanomaterials 2016, 8910271. 10.1155/2016/8910271

[B41] Mulla S. I. Ameen F. Talwar M. P. Shah Eqani SAMA Bharagava R. N. Saxena G. (2020). “Organophosphate pesticides: impact on environment, toxicity, and their degradation,” in Bioremediation of industrial waste for environmental safety (Singapore, Singapore: Springer), 265–290.

[B42] Musah M. Azeh Y. Mathew J. Umar M. Abdulhamid Z. Muhammad A. (2022). Adsorption kinetics and isotherm models: a review. Caliphate J. Sci. Technol. 4 (1), 20–26. 10.4314/cajost.v4i1.3

[B43] Ore O. T. Adeola A. O. Bayode A. A. Adedipe D. T. Nomngongo P. N. (2023). Organophosphate pesticide residues in environmental and biological matrices: occurrence, distribution and potential remedial approaches. Environ. Chem. Ecotoxicol. 5, 9–23. 10.1016/j.enceco.2022.10.004

[B44] Pan L. Sun J. Li Z. Zhan Y Xu S. Zhu L. (2018). Organophosphate pesticide in agricultural soils from the yangtze river delta of China: concentration, distribution, and risk assessment. Environ. Sci. Pollut. Res. 25 (1), 4–11. 10.1007/s11356-016-7664-3 27687760

[B45] Sagar V. Kukkar D. (2023). Facile adsorption of organophosphate pesticides over HKUST-1 MOFs. Environ. Monit. Assess. 195 (9), 1056. 10.1007/s10661-023-11662-3 37592149

[B46] Sarno M. Casa M. Cirillo C. Ciambelli P. (2017). Complete removal of persistent pesticide using reduced graphene oxide-silver nanocomposite. Chem. Eng. Trans. 60, 151–156. 10.3303/CET1760026

[B47] Seif M. Mohamed M. S. Khalil F. A. Assem A. K. A. A. Abou Donia M. A. Mohamed S. R. (2015). The adsorptive capacity of activated carbon and its nano-particles in removal of organophosphorus malathion from aqueous solution. J. Agroaliment. Processes Technol. 21, 116–124.

[B48] Sereshti H. Amirafshar A. Kadi A. Hamid R. N. Rezania S. Hoang H. Y. (2023). Isolation of organophosphate pesticides from water using gold nanoparticles doped magnetic three-dimensional graphene oxide. Chemosphere 320, 138065. 10.1016/j.chemosphere.2023.138065 36754307

[B49] Skopp J. (2009) Derivation of the Freundlich adsorption isotherm from kinetics. (San Diego, CA: ACS). Available at: http://www.jce.divched.org/Journal/Issues/2009/Nov/abs1341.html.

[B50] Sumon K. A. Rashid H. Peeters E. T. H. M. Bosma R. H. Paul J. V. B. (2018). Environmental monitoring and risk assessment of organophosphate pesticides in aquatic ecosystems of North-west Bangladesh. Chemosphere 206, 92–100. 10.1016/j.chemosphere.2018.04.167 29734095

[B51] Thamer B. M. El-Hamshary H. Al-Deyab S. S. El-Newehy M. H. (2019). Functionalized electrospun carbon nanofibers for removal of cationic dye. Arabian J. Chem. 12 (6), 747–759. 10.1016/j.arabjc.2018.07.020

[B52] Uddin S. (2021). Removal of pesticides using carbon-based nanocomposite materials, in Green energy and technology (Berlin, Germany: Springer Science and Business Media Deutschland GmbH), 365–385.

[B53] Velasco A. Hernández S. Ramírez M. Ortíz I. (2014). Detection of residual organochlorine and organophosphorus pesticides in agricultural soil in rio verde region of san luis potosi, Mexico. J. Environ. Sci. Health. Part B. 49 (7), 498–504. 10.1080/03601234.2014.896670 24813984

[B54] Wanjeri V. W. O. Sheppard C. J. Prinsloo A. R. E. Ngila J. C. Ndungu P. G. (2018). Isotherm and kinetic investigations on the adsorption of organophosphorus pesticides on graphene oxide based silica coated magnetic nanoparticles functionalized with 2-phenylethylamine. J. Environ. Chem. Eng. 6 (1), 1333–1346. 10.1016/j.jece.2018.01.064

[B55] Yang L. Zhang X. Jiang L. (2019). Determination of organophosphorus pesticides in fortified tomatoes by fluorescence quenching of cadmium selenium–zinc sulfide quantum dots. Anal. Lett. 52 (5), 729–744. 10.1080/00032719.2018.1490311

[B56] Yin Z. Allado K. Sheardy A. T. Ji Z. Arvapalli D. Liu M. (2021). Mingled MnO2and Co3O4Binary nanostructures on well-aligned electrospun carbon nanofibers for nonenzymatic glucose oxidation and sensing. Cryst. Growth. Des. 21 (3), 1527–1539. 10.1021/acs.cgd.0c01299

[B57] Yin Z. Ji Z. Bloom B. P. Jayapalan A. Liu M. Zeng X. (2022). Manipulating cobalt oxide on N-doped aligned electrospun carbon nanofibers towards instant electrochemical detection of dopamine secreted by living cells. Appl. Surf. Sci. 577, 151912. 10.1016/j.apsusc.2021.151912

[B58] Zeng Z. Liu Y. Zhang W. Chevva H. Jianjun W. (2017). Improved supercapacitor performance of MnO2-electrospun carbon nanofibers electrodes by MT magnetic field. J. Power. Sources 358, 22–28. 10.1016/j.jpowsour.2017.05.008

[B59] Zhang L. Aboagye A. Kelkar A. Lai C. Fong H. (2014). A review: carbon nanofibers from electrospun polyacrylonitrile and their applications. J. Mater. Sci. 49, 463–480. 10.1007/s10853-013-7705-y

[B60] Zhang W. miao Yan J. Su Q. Han J. Gao J. F (2022). Hydrophobic and porous carbon nanofiber membrane for high performance solar-driven interfacial evaporation with excellent salt resistance. J. Colloid. Interface Sci. 612, 66–75. 10.1016/j.jcis.2021.12.093 34974259

[B61] Zhang Y. Pan Q. Lu S. Liu X. Zhai J. Xu J. (2021). Occurrence and risk evaluation of organophosphorus pesticides in typical water bodies of Beijing, China. Environ. Sci. Pollut. Res. 28 (2), 1454–1463. 10.1007/s11356-020-10288-z 32839911

[B62] Zhou Z. Liu K. Lai C. Zhang L. Li J. Hou H. (2010). Graphitic carbon nanofibers developed from bundles of aligned electrospun polyacrylonitrile nanofibers containing phosphoric acid. Polymer 51 (11), 2360–2367. 10.1016/j.polymer.2010.03.044

[B63] Zhu J. Yu J. Zhang B. Li C. Wang J. Ji J. (2023). Hydrophobic-action-driven removal of six organophosphorus pesticides from tea infusion by modified carbonized bacterial cellulose. Food. Chem. 412, 135546. 10.1016/j.foodchem.2023.135546 36716625

[B64] Zhu X. Li B. Yang J. Li Y. Zhao W. Shi J. (2015). Effective adsorption and enhanced removal of organophosphorus pesticides from aqueous solution by Zr-based MOFs of UiO-67. ACS. Appl. Mater. Interfaces 7 (1), 223–231. 10.1021/am5059074 25514633

[B65] Zussman E. Chen X. Ding W. Calabri L. Dikin D. A. Quintana J. P. (2005). Mechanical and structural characterization of electrospun PAN-derived carbon nanofibers. Carbon 43 (10), 2175–2185. 10.1016/j.carbon.2005.03.031

