# Supplementary material for: Adsorptive removal of organophosphate pesticides from aqueous solution using electrospun carbon nanofibers
Source: Front Chem. 2024 Aug 26;12:1454367. doi: 10.3389/fchem.2024.1454367 (PMC11381308; doi:10.3389/fchem.2024.1454367)
Supplement: Supplementary file 1 [file DataSheet1.PDF]

Supplementary Material

Adsorptive Removal of Organophosphate Pesticides from Aqueous Solution Using Electrospun Carbon Nanofibers

Bukola O Adesanmi[1], Shobha Mantripragada[2], Raphael D. Ayivi[1], Panesun Tukur[1], Sherine O. Obare[1,2]*, Jianjun Wei[1]*

[1]Department of Nanoscience, Joint School of Nanoscience and Nanoengineering, University of North Carolina, Greensboro, NC 27412, USA

[2]Department of Nanoengineering, Joint School of Nanoscience and Nanoengineering, North Carolina Agricultural and Technical State University, Greensboro, NC 27411, USA

***Correspondence:**

Jianjun Wei

j_wei@uncg.edu

Sherine Obare

soobare@uncg.edu

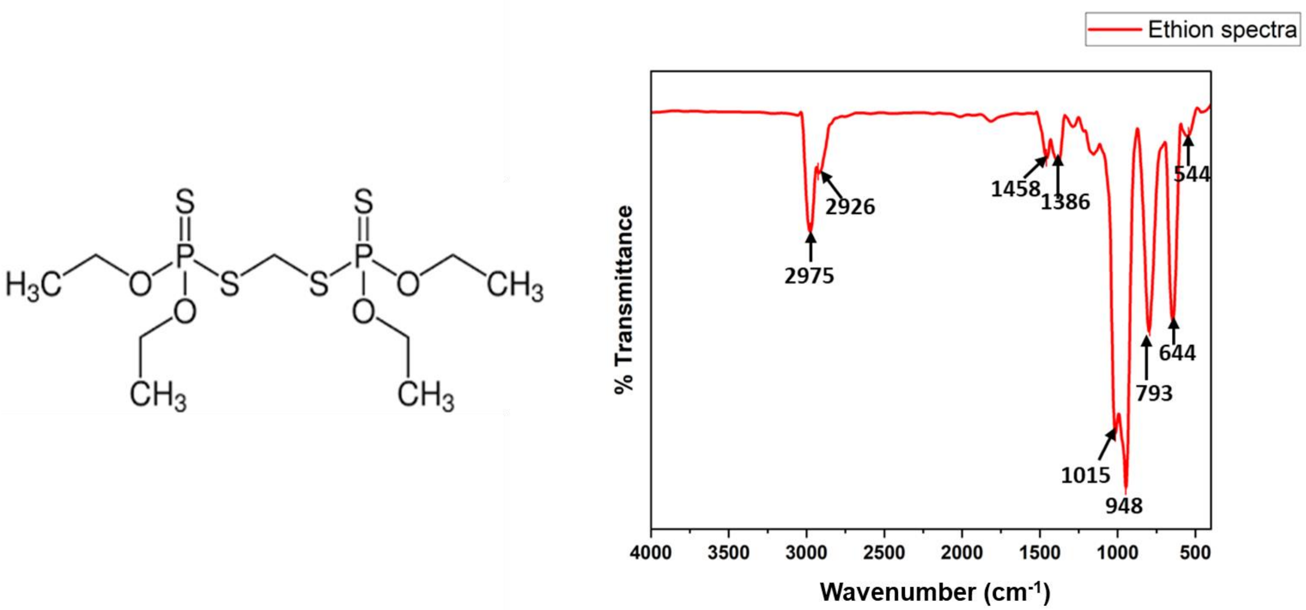

Figure S1: Chemical structure and FTIR spectra of ethion pesticide.

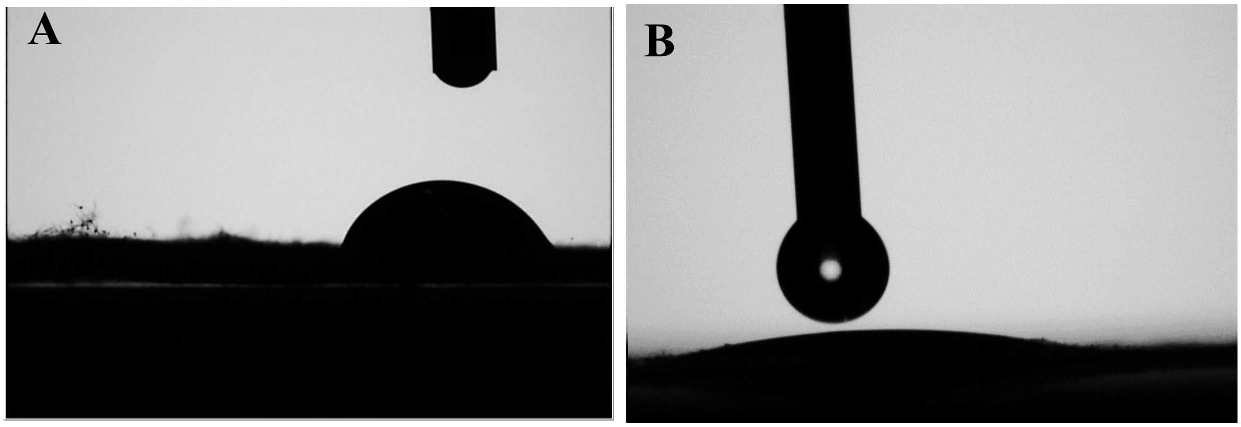

Figure S2: Contact angle images of (a) R-ECNFs (a) A-ECNFs

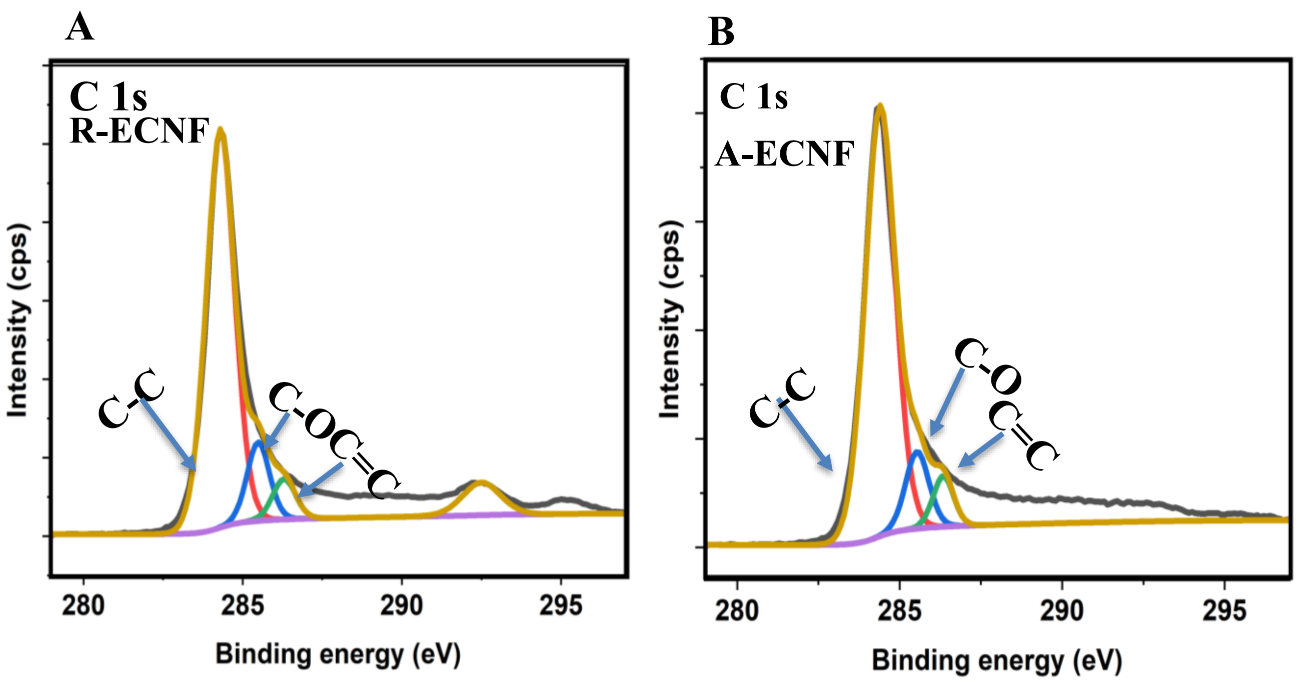

Figure S3: C1s elemental scan of (a)R-ECNFs and (b)A-ECNFs fibers.

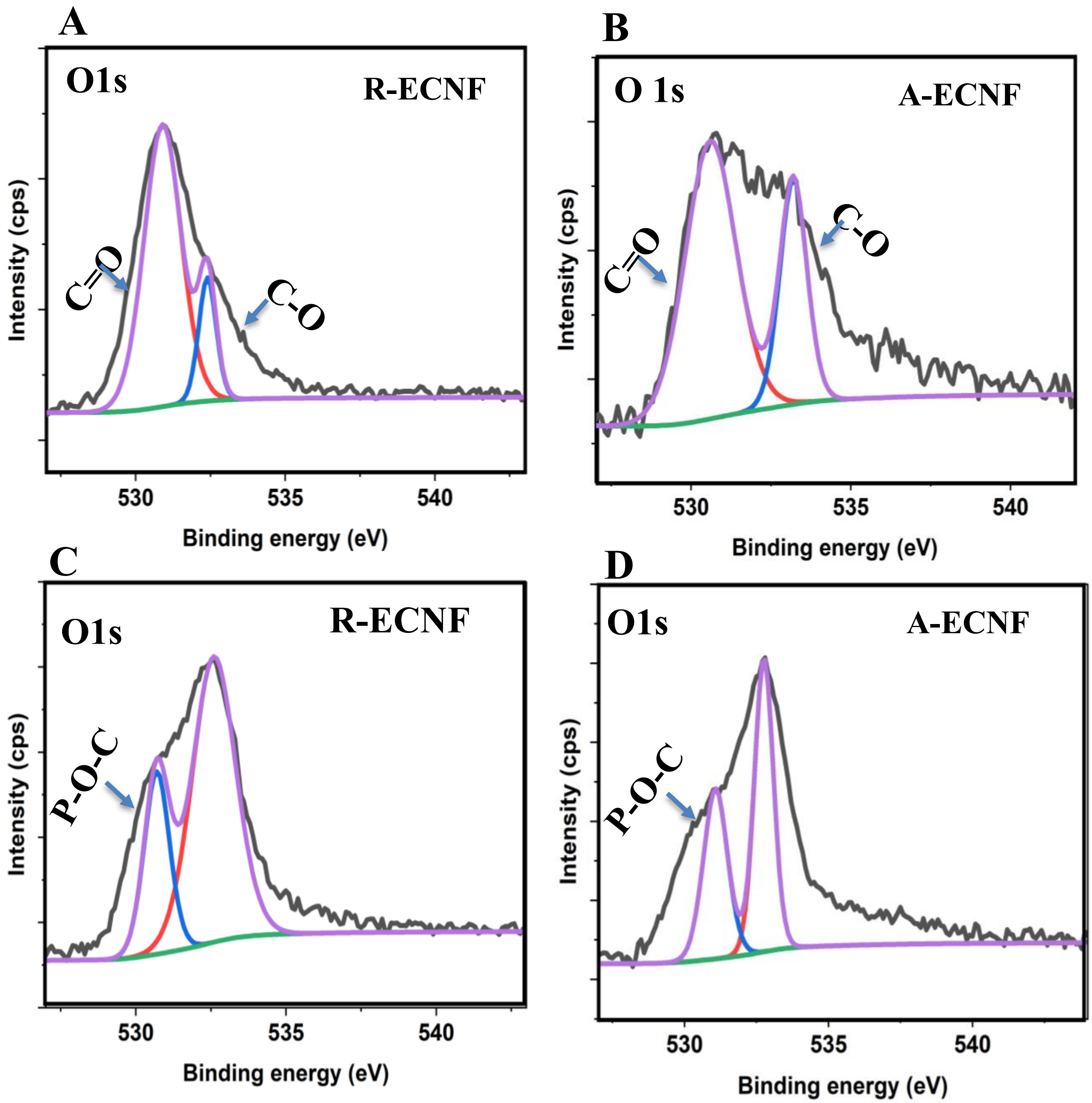

Figure S4: O 1s elemental scan of random and aligned fibers (a) and (b)before adsorption; (c) and (d)after adsorption

Table S1: Atomic composition of R-ECNF and A-ECNF before and after adsorption.

	Before adsorption		After adsorption	
Element	R-ECNF	A-ECNF	R-ECNF	A-ECNF
Carbon	93.5	93.4	90.6	93.1
Oxygen	4.8	4.4	5.4	4.8
Nitrogen	1.8	1.6	1.8	0.7
Phosphorus	-	-	0.8	0.4
Sulphur	-	-	1.3	0.7

Table S2: Percentage of detected phosphorus and Oxygen chemical bond before and after adsorption by XPS

	Before Adsorption		After adsorption	
	R-ECNF	A-ECNF	R-ECNF	A-ECNF
C=O	82.8	69.9	72.8	57.3
C-O	17.2	30.1	27.2	42.7
$2P_{3/2}$	-	-	89.9	46.8
$2P_{1/2}$	-	-	-	29
P-O-C			10.1	24.2

RCNF after adsorption Phosphorus XPS information

Name	Start BE	Peak BE	End BE	Height CPS	FWHM eV	Area (P) CPS.eV	Area (N) TPP-2M	Atomic %
P2p Scan A	143.9	133.4	124.1	1357.07	1.08	1583.74	0.01	89.89
P2p Scan B	143.9	134.12	124.1	449.63	0.37	178.03	0	10.11

ACNF after adsorption Phosphorus XPS information

Name	Start BE	Peak BE	End BE	Height CPS	FWHM eV	Area (P) CPS.eV	Area (N) TPP-2M	Atomic %
P2p Scan A	143.9	133.1	124.1	601.1	0.56	363.28	0	46.78
P2p Scan B	143.9	133.67	124.1	515.3	0.41	225.51	0	29.04
P2p Scan C	143.9	134.3	124.1	360.33	0.49	187.73	0	24.18

RCNF XPS survey after adsorption

Name	Start BE	Peak BE	End BE	Height CPS	FWHM eV	Area (P) CPS.eV	Area (N) TPP-2M	Atomic %
P 2p	144	133.25	126.43	9031.56	3.9	38530.16	0.03	0.82
S 2p	175	162.79	157	18399.5	4.26	86006.03	0.05	1.34
C 1s	298	284.27	279	597066.16	4.18	2923743.95	3.32	90.64
N 1s	410	400.41	392	16426.89	4.86	91890.47	0.07	1.81
O1s	540.36	531.95	525	89616.71	4.57	434764.93	0.2	5.4

ACNF XPS survey after adsorption

Name	Start BE	Peak BE	End BE	Height CPS	FWHM eV	Area (P) CPS.eV	Area (N) TPP-2M	Atomic %
P 2p	144	133.08	125.25	3725.76	4.11	17679.61	0.01	0.35
S 2p	175	162.86	157	10100.45	4.27	49291.2	0.03	0.71
C 1s	298	284.24	279	647420.73	4.18	3271769.35	3.72	94.12
N 1s	408.72	400.54	392.32	7503.42	4.84	40146.86	0.03	0.73
O1s	545	531.88	525	63361.35	5.1	354376.96	0.16	4.08
